# Cardiovascular system characteristics in HIV/AIDS patients with talaromycosis

**DOI:** 10.1590/1414-431X2025e14655

**Published:** 2026-02-16

**Authors:** Lanwei Nong, Jie Ma, Yanqing Zheng, Qingdong Zhu, Cailing Wei, Jieling Chen, Sijun Li

**Affiliations:** 1 Infectious Disease Laboratory, Guangxi AlDS Clinical Treatment Center (Nanning), The Fourth People's Hospital of Nanning, Nanning, Guangxi, China; 2 Department of Surgery, Guangxi AlDS Clinical Treatment Center (Nanning), The Fourth People's Hospital of Nanning, Nanning, Guangxi, China; 3 Department of Tuberculosis, Guangxi AlDS Clinical Treatment Center (Nanning), The Fourth People's Hospital of Nanning, Nanning, Guangxi, China; 4 Department of Internal Medicine, Guangxi AlDS Clinical Treatment Center (Nanning), The Fourth People's Hospital of Nanning, Nanning, Guangxi, China

**Keywords:** Systemic mycosis, Cardiac remodeling, Opportunistic cardiac complications, Fungal cardiomyopathy, Immunodeficiency-associated cardiovascular disease

## Abstract

Patients with acquired immune deficiency syndrome (AIDS) are more vulnerable to opportunistic infections (OIs) such as *Talaromycosis marneffei* (TSM), which is associated with a high mortality rate. Nevertheless, the effect of TSM on the cardiovascular system of affected patients remains elusive. To that end, this research aimed to investigate the impact of TSM on the cardiovascular system in the individuals with HIV/AIDS. Participants were assigned to the HIV/AIDS patients group or the HIV/AIDS patients with TSM (HIV/AIDS+TSM) group. A total of 120 individuals were included in the present study, with 59 in the HIV/AIDS group and 61 in the HIV/AIDS+TSM group. Myocardial serum markers, color Doppler cardiovascular ultrasound (CCU), and 24-h ambulatory electrocardiograph (AECG) data were collected and analyzed for both groups. Compared with the HIV/AIDS group, the HIV/AIDS+TSM group exhibited a significant increase in left and right diameters of the right atrium (LR-RA) and right ventricle (LR-RV), while the anteroposterior diameter of the right ventricle outflow tract (AT-RVOT), the interventricular septal thickness (IVS), and the left ventricular posterior wall thickness (LVPW) were significantly reduced. The HIV/AIDS+TSM group showed higher AECG abnormality rates, particularly for non-sinus rhythms and ST-T changes. Our findings demonstrated significant cardiac functional alterations in HIV/AIDS patients with TSM co-infection compared to HIV/AIDS alone, underscoring the necessity for enhanced cardiovascular monitoring in this vulnerable population.

## Introduction

Acquired immune deficiency syndrome (AIDS) is a collection of symptoms induced by the human immuno-deficiency virus (HIV). The world continues to grapple with the HIV epidemic, with 1.3 million new infections reported in 2022 (1). Of note, the introduction of the highly active antiretroviral therapy (HAART) into clinical practice has significantly reduced the mortality rate of HIV/AIDS patients (2). In addition to the immune system, HIV can affect multiple systems (3,4). Although HAART has extended the life expectancy of HIV/AIDS patients to near-normal levels, there is still a high incidence of nervous, cardiovascular, and endocrine system-related disorders, often with early onsets that can negatively impact the quality of life of affected patients (5). Despite the effectiveness of HAART in reducing the incidence of opportunistic infections (OIs) in HIV/AIDS patients, the rate of OIs remains at 4.9% (6). Importantly, these OIs can lead to dysfunction in multiple systems and negatively impact prognosis (7). Cardiovascular dysfunction, especially heart failure, is a major risk factor for poor outcomes in HIV/AIDS patients with OIs (8).

The main pathogenic microorganisms responsible for OIs in HIV/AIDS patients are *Mycobacterium tuberculosis* (Mtb), *Cryptococcus neoformans,* and *Talaromyces marneffei* (TSM), which causes talaromycosis (9). Among these, OIs caused by TSM have the highest mortality rate, affecting up to 16.2% of patients with talaromycosis. TSM, also known as *Penicillium marneffei* is a common pathogenic microbe in Southeast Asia. It is a thermally dimorphic fungus that exists as isolated mycelium at 25°C and transforms into a pathogenic yeast form at 37°C (10). TSM infects the host through four main steps: adhering to and colonizing host tissue, multiplying, escaping, or destroying the host defense system, and damaging the host tissue (11). The host's cellular immunity, particularly the T-cell-mediated Th1 response, is crucial in resisting TSM invasion (12). TSM primarily invades the human mononuclear-macrophage system and is more likely to infect immunocompromised individuals (13).

Systemic fungal infections are increasingly recognized as contributors to cardiovascular dysfunction through both direct invasion and systemic inflammatory effects (14). While TSM infection causes granulomatous inflammation in multiple organs (15), its cardiac-specific manifestations remain poorly characterized, although clinical evidence exists, such as case reports of TSM-associated pericardial effusions (16). In HIV/AIDS patients - who already face elevated cardiovascular risk due to chronic inflammation and immune dysfunction - TSM co-infection may significantly exacerbate cardiac pathology. This interaction carries particular clinical importance in endemic regions like Nanning, China, where subtropical monsoon climates favor TSM transmission. To address these critical knowledge gaps, we performed the first comprehensive comparative analysis of cardiovascular parameters in HIV patients with and without TSM co-infection, including: 1) systematic evaluation of myocardial injury markers; 2) detailed echocardiographic assessment of cardiac structure/function; and 3) ambulatory ECG monitoring for arrhythmia detection. This study provides essential baseline data to guide cardiac monitoring and management in this high-risk population, while identifying specific cardiovascular signatures of TSM co-infection that warrant further investigation.

## Material and Methods

### Study design

Data from patients diagnosed with HIV/AIDS with or without TSM infection, at the Fourth People's Hospital of Nanning City from January 2022 to December 2023 were retrieved from hospital records for analysis. The study protocol was reviewed and approved by the Hospital's Institutional Ethics Committee (approval number [2021] 25). The inclusion criteria were as follows: i) Patients in the HIV/AIDS group were diagnosed with HIV/AIDS and did not have OIs. All the patients were in the AIDS stage (HIV positive with CD4 counts <200 or HIV positive with OIs); ii) Patients in the HIV/AIDs+TSM group were diagnosed with HIV/AIDS combined with TSM infection only. A diagnosis of TSM infection was confirmed through blood cultures, bone marrow, or body fluids that tested positive for TSM. iii) The patient or their attendants gave signed informed consent. The exclusion criteria were as follows: i) patients aged 65 years or older; ii) patients with a previous history of cardiovascular diseases (such as hypertension, coronary heart disease, myocardial infarction, myocarditis, pulmonary hypertension, dilated cardiomyopathy, valvular heart disease, ischemic heart disease, etc.); iii) patients with metabolic diseases (such as diabetes, hyperthyroidism, Cushing's syndrome, primary hyperaldosteronism, etc); iv) patients with malignancies; v) patients affected by other pathogenic microorganisms; vi) patients admitted to the ICU; and vii) patients without signed consent. A total of 120 individuals were included in the present study, with 59 in the HIV/AIDS group and 61 in the HIV/AIDS+TSM group.

### Clinical data collection

Clinical data collected at baseline following admission included: i) disease course (number of days since testing positive for HIV); ii) height, weight, and body mass index; iii) CD4 T-cell counts; iv) antiretroviral treatment; v) cardiovascular risk factors, including smoking and alcohol history; vi) blood lipids, including triglycerides (TG) (reference value: 0-1.7 mmol/L), total cholesterol (TC) (reference value: 0-5.18 mmol/L), high-density lipoprotein cholesterol (HDL) (reference value: 1.04-1.66 mmol/L); low-density lipoprotein cholesterol (LDL) (reference value: 0-3.37 mmol/L); vii) serum myocardial enzyme levels, including creatine kinase (CK) (reference value: 50-310 U/L), creatine kinase-MB (CK-MB) (reference value: 0-25 U/L), lactic dehydrogenase (LDH) (reference value: 120-250 U/L), α-hydroxybutyrate dehydrogenase (α-HBD) (reference value: 72-182 U/L), troponin-I (Tn-I) (reference value: 0-40 ng/L); viii) color cardiac ultrasound (CCU), including aortic sinus diameter (AO) (reference value: 25-35 mm), ascending aorta inner diameter (AAO) (reference value: 25-35 mm), main pulmonary artery (MPA) diameter (reference value: 18-24 mm), left-right diameter of the right atrium (LR-RA) (reference value: 25-35mm), transverse diameter of RA (TR-RA), left-right diameter of the right ventricle (LR-RV) (reference value: 20-35 mm), anteroposterior diameter of RV (AT-RV) (reference value: <23 mm), right ventricle outflow tract (RVOT), anteroposterior diameter of RVOT (reference value: 20-30 mm), interventricular septal thickness (IVS) (reference value: 8-12 mm), anteroposterior diameter of the left atrium (RT-LA) (reference value: 20-35 mm), anteroposterior diameter of the left ventricle end-diastolic-radial-line (AT-LV-D) (reference value: 35-40 mm), anteroposterior diameter of the left ventricle end-systolic-radial-line (AT-LV-S) (reference value: 20-35 mm), left ventricular posterior wall thickness (LVPW) (reference value: 9-11 mm), ejection factions (EF) (reference value: >50%), fractional shortening (FS) (reference value: >25%); and 9) 24-h ambulatory electrocardiograph (AECG). The participants were categorized into normal AECG and abnormal AECG based on diagnostic findings, with heart rate (HR) simultaneously recorded.

### Statistical analysis

To compare the differences between two independent groups, the appropriate statistical tests were selected based on data distribution and variance assumptions. If the data followed a normal distribution (assessed using Shapiro-Wilk or Kolmogorov-Smirnov tests) and met homogeneity of variance (Levene's test), an independent samples *t*-test was applied. For non-normally distributed data, the Mann-Whitney U test (a non-parametric alternative) was used. When the data met normality assumptions but exhibited heteroscedasticity (unequal variances), Welch's *t*-test was used. Categorical variables (e.g., ECG abnormalities) are reported as counts (%) and compared using chi-squared tests or Fisher's exact tests (if expected frequencies <5). Odds ratios (ORs) with 95% confidence intervals (CIs) quantified association strengths. Subgroup analyses examined specific ECG abnormalities (rhythm, ST-T changes, QT prolongation). All tests were two-tailed, with statistical significance set at a P value <0.05. All statistical analyses were performed using SPSS software (SPSS, version 25.0, IBM, USA). All graphs were generated using GraphPad Prism software (GraphPad, version 9.0, USA).

## Results

### Baseline characteristics

**Figure 1:**
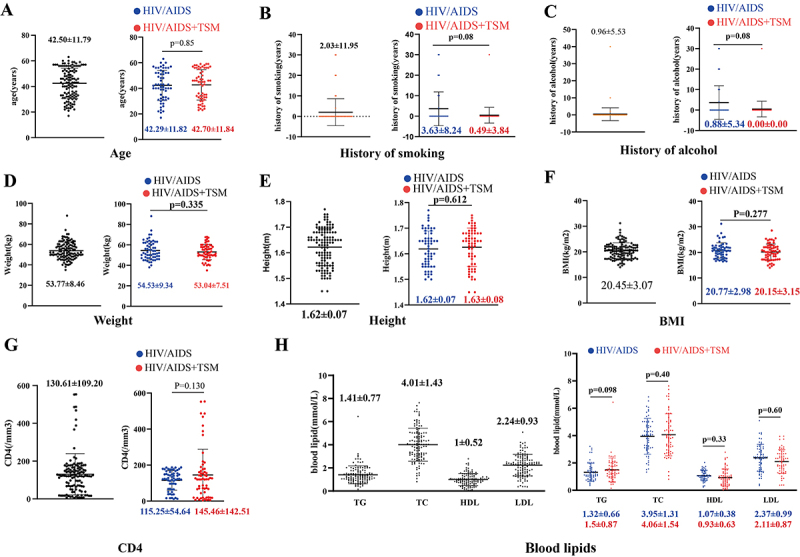
Baseline characteristics of all patients divided into HIV/AIDS group or HIV/AIDS with *Talaromycosis marneffei* infection (HIV/AIDS+TSM) group. **A**, Age distribution; **B**, history of smoking; **C**, history of alcohol; **D**, weight distribution; **E**, height distribution; **F**, BMI distribution; **G**, CD4 distribution; **H**, blood lipid distribution. Data are reported as medians and interquartile range (Mann-Whitney U test). BMI: body mass index; TG: triglycerides; TC: total cholesterol; HDL: high-density lipoprotein cholesterol; LDL: low-density lipoprotein cholesterol.

A total of 120 individuals were included in the present study, with 59 in the HIV/AIDS group and 61 in the HIV/AIDS+TSM group, comprising 93 men and 27 women. The baseline characteristics of all patients are detailed in Table 1 and Figure 1. Since patients with cardiovascular diseases, metabolic disorders, and hyperlipidemia were excluded, we assessed cardiovascular risk in the two groups by comparing their smoking history, alcohol history, and blood lipids. Our analysis revealed that the baseline risk of cardiovascular disease was not higher in the HIV/AIDS+TSM group compared to the HIV/AIDS group (Figure 1). There were no significant differences between the two groups in terms of age, disease course, smoking history, alcohol consumption history, or blood lipid profiles.

**Table 1:** Baseline characteristics of all patients and patients divided into HIV/AIDS group or HIV/AIDS with *Talaromycosis marneffei* infection (HIV/AIDS+TSM) group.

Population characteristics	All patients	HIV/AIDS	HIV/AIDS+TSM
Disease course, days, mean±SD	194.87±613.79	147.31±504.84	240.87±704.60 (*vs* HIV/AIDS, P=0.41)
Male	93	40	53
Female	27	19	8
Antiretroviral treatment			
3TC+AZT+NVP	10	10	0
3TC+LPV/r	5	5	0
ANV+TDF+3TC	6	0	6
AZT+3TC+EFV	17	6	11
AZT+3TC+LPV/r	3	0	3
Biktarvy	12	7	5
DTG/3TC	5	5	0
NVP+TDF+3TC	3	3	0
TDF+3TC+EFV	48	20	28
TDF+3TC+LPV/r	11	3	8
Individuals with a history of smoking	18	14	4
Individuals with a history of alcohol	7	7	0

Data (except disease course) are reported as number. BMI: body mass index; ANV: Ainuovirine; TDF: Tenofovir disoproxil fumarate; DTG: Dolutegravir; 3TC: Lamivudine; EFV: Efavirenz; AZT: Zidovudine; LPV/r: Lopinavir/ritonavir; NVP: Nevirapine.

### Differences in cardiovascular characteristics between the HIV/AIDS and HIV/AIDS+TSM groups

**Figure 2:**
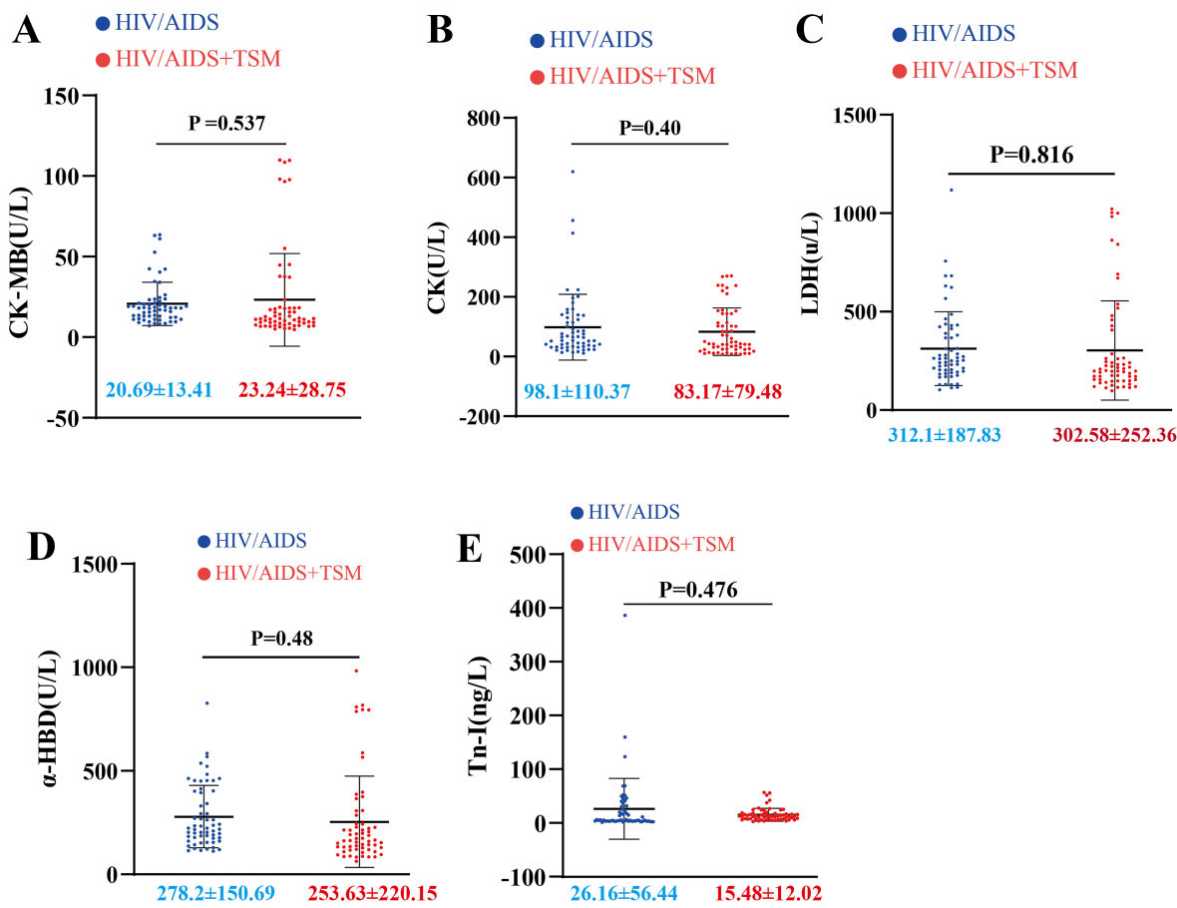
Myocardial enzyme profiles of patients divided into HIV/AIDS group or HIV/AIDS with *Talaromycosis marneffei* infection (HIV/AIDS+TSM) group. **A**, Comparison of CK-MB levels between the two groups. **B**, CK levels. **C**, LDH levels. **D**, αHBD levels. **E**, Tn-I levels. Data are reported as medians and interquartile range (Mann-Whitney U test). CK: creatine kinase; CK-MB: creatine kinase-MB; LDH: lactic dehydrogenase; α-HBD: α-hydroxybutyrate dehydrogenase; Tn-I: troponin-I.

**Figure 3:**
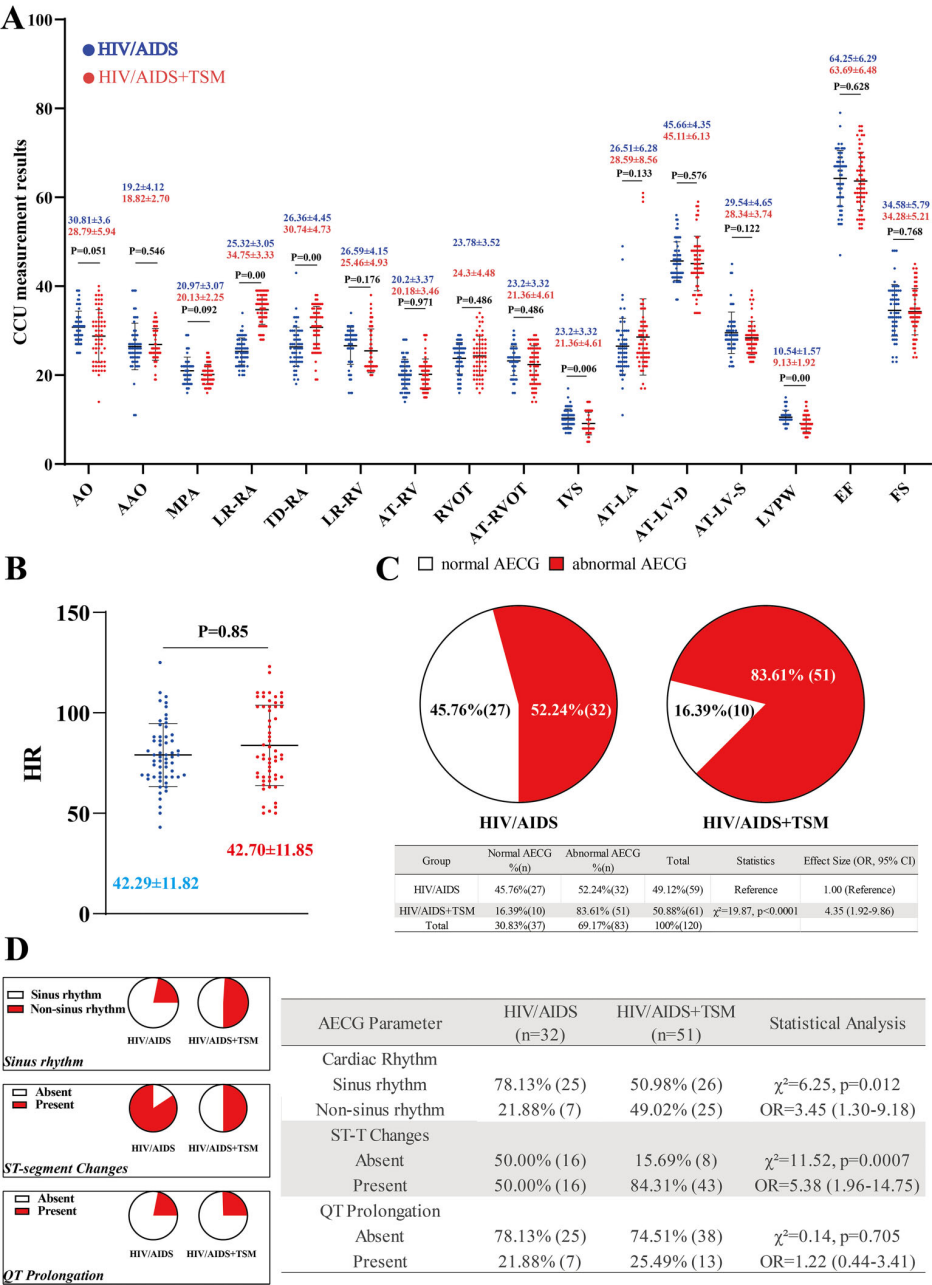
Color Doppler cardiovascular ultrasound (CCU) measurements and electrocardiograph analyses of patients divided into HIV/AIDS group or HIV/AIDS with *Talaromycosis marneffei* infection (HIV/AIDS+TSM) group. **A**, CCU measurement. **B**, Heart rate (HR). Data are reported as medians and interquartile range (Mann-Whitney U test). **C**, Comparison of ambulatory electrocardiograph (AECG) abnormalities between the two groups. **D**, Detailed analysis of abnormal AECG patterns. AO: aortic sinus diameter; AAO: ascending aorta inner diameter; MPA: main pulmonary artery diameter; RA: right atrium; LR-RA: left-right diameter of RA; TD-RA: transverse diameter of RA; RV: right ventricle; LR-RV: left-right diameter of RV; AT-RV: anteroposterior diameter of RV; RVOT: right ventricle outflow tract; AT-RVOT: anteroposterior diameter of RVOT; IVS: interventricular septal thickness; LA: left atrium; AT-LA: anteroposterior diameter of LA; LV: left ventricle; AT-LV-D: anteroposterior diameter of LV end-diastolic-radial-line; AT-LV-S: anteroposterior diameter of LV end-systolic-radial-line; LVPW: left ventricular posterior wall thickness; EF: ejection fraction; FS: fractional shortening.

Continuous variables are described using medians and interquartile ranges (Table 2). The myocardial enzyme profiles are shown in Figure 2. No statistically significant differences in myocardial enzyme profiles were observed between the HIV/AIDS and HIV/AIDS+TSM groups (Figures 2A-E). The results of CCU measurement and AECG are presented in Figure 3. Compared with the HIV/AIDS group, the LR-RA and LR-RV were significantly increased in the HIV/AIDS+TSM group, while the IVS and LVPW were significantly decreased (Figure 3A). No significant difference was observed in heart rate between the HIV/AIDS and HIV/AIDS+TSM groups (Figure 3B). The prevalence of ECG abnormalities was significantly higher in HIV/AIDS+TSM patients (83.61%, 51/61) compared to HIV/AIDS patients (54.24%, 32/59) (χ^2^=19.87, P<0.0001; OR=4.35, 95%CI:1.92-9.86) (Figure 3C). Detailed analysis of abnormal AECG patterns revealed that HIV/AIDS+TSM patients showed higher prevalence of non-sinus rhythms (49.02% [25/51]) than HIV/AIDS alone (21.88% [7/32]) (OR=3.45, 95%CI:1.30-9.18; P=0.012), and marked ST-T abnormalities were more frequent in HIV/AIDS+TSM (84.31% [43/51]) compared to HIV/AIDS (50.00% [16/32]) (OR=5.38, 95%CI:1.96-14.75; P<0.001) (Figure 3D). No significant difference in QT prolongation was observed between HIV/AIDS+TSM (25.49% [13/51]) and HIV/AIDS (21.88% [7/32]) (P=0.705) groups.

**Table 2:** Medians and interquartile ranges of the continuous variables (n=120).

Variables	Median	Minimum	Maximum	Percentiles	
				25%	75%
Age	43.00	17.00	63.00	32.25	53.75
Disease course, days	30.00	1.00	3650.00	14.00	120.00
Height, m	1.64	1.45	1.77	1.56	1.68
Weight, kg	52.00	35.00	88.00	48.00	59.85
BMI, kg/m^2^	20.53	14.02	31.18	17.97	22.06
TG, mmol/L	1.24	0.15	6.44	0.95	1.76
TC, mmol/L	4.02	0.81	7.63	3.02	5.00
HDL, mmol/L	0.99	0.03	2.79	0.67	1.30
LDL, mmol/L	2.32	0.36	5.09	1.52	2.83
CD4, /mm^3^	121.50	4.00	553.00	64.25	165.00
CK-MB, U/L	14.70	4.79	109.90	9.60	20.89
CK, U/L	52.55	7.70	619.60	30.93	128.88
LDH, U/L	226.00	98.00	1118.00	171.28	376.68
α-HBD, U/L	201.20	63.00	983.70	142.20	324.98
Tn-I, ng/L	11.36	1.04	386.06	4.60	20.00
AO, mm	30.00	14.00	40.00	27.00	32.75
AAO, mm	18.50	11.00	28.00	16.25	21.00
MPA, mm	26.00	11.00	39.00	24.00	29.00
LR-RA, mm	20.00	16.00	29.00	18.00	22.00
Tr-RA, mm	29.00	20.00	39.00	25.00	36.00
LR-RV, mm	28.50	18.00	43.00	25.00	33.00
AT-RV, mm	26.50	16.00	38.00	22.00	29.00
RVOT, mm	20.00	14.00	29.00	17.25	22.00
AT-RVOT, mm	25.00	16.00	34.00	21.00	27.00
IVS, mm	23.00	13.00	30.00	19.00	26.00
RT-LA, mm	9.50	5.00	17.00	8.00	12.00
AT-LV-D, mm	26.00	11.00	61.00	23.00	30.00
AT-LV-S, mm	45.00	34.00	59.00	42.00	48.00
LVPW, mm	28.00	22.00	45.00	26.00	30.00
EF, %	10.00	6.00	15.00	9.00	11.00
FS, %	63.50	47.00	79.00	60.00	69.00
HR	78.00	43.00	125.00	68.00	95.75

BMI: body mass index; TG: triglyceride; TC: total cholesterol; HDL: high-density lipoprotein cholesterol; LDL: low-density lipoprotein cholesterol; CK: creatine kinase; LDH: lactic dehydrogenase; α-HBD: α-hydroxybutyrate dehydrogenase; Tn-I: troponin-I; AO: aortic sinus diameter; AAO: ascending aorta inner diameter; MPA: main pulmonary artery diameter; LR-RA: left-right diameter of the right atrium; Tr-RA: transverse diameter of the right atrium; LR-RV: left-right diameter of the right ventricle; AT-RV: anteroposterior diameter of the right ventricle; RVOT: right ventricle outflow tract; AT-RVOT: anteroposterior diameter of the right ventricle outflow tract; IVS: interventricular septal thickness; RT-LA: anteroposterior diameter of the left atrium; AT-LV-D: anteroposterior diameter of the left ventricle end-diastolic; AT-LV-S: anteroposterior diameter of the left ventricle end-systolic; LVPW: left ventricular posterior wall thickness; EF: ejection fraction; FS: fractional shortening; HR: heart rate.

## Discussion

Beyond immune system impairment, individuals with HIV/AIDS may experience impairments in multiple systems (3,4). Among these, cardiovascular damage can have severe consequences, with conditions such as acute coronary syndrome (ACS) and Takotsubo syndrome being life-threatening emergencies (17). Despite the widespread use of HARRT, OIs are still significantly prevalent in HIV/AIDS patients (6). OIs not only increase the risk of cardiovascular diseases (18), but also render HIV/AIDS patients more susceptible to heart failure (8). TSM infection, which carries a high mortality rate, is recognized as one of the most opportunistic infections in HIV/AIDS patients (9,19). Following TSM infection, the primary manifestations include dysfunction and tissue damage in the respiratory organs, digestive organs, and skin (20). However, there are no relevant reports on the effects of TSM on the cardiovascular system, particularly in HIV/AIDS patients.

In this study, we analyzed clinical data related to the cardiovascular health of HIV/AIDS patients and HIV/AIDS patients with TSM infection. We found no significant differences in age or disease course between the HIV/AIDS group and the HIV/AIDS+TSM group, suggesting that these factors did not influence the outcomes. Moreover, there were no significant differences in myocardial enzyme levels between the two groups, indicating that TSM did not significantly damage cardiomyocytes. Importantly, based on the results of CCU, significant differences were observed in the LR-RA and LR-RV, AT-RVOT, IVS, and LVPW between the HIV/AIDS and HIV/AIDS+TSM groups. Although the LR-RA and LR-RV were not significantly higher than the reference values, they were significantly higher in the HIV/AIDS+TSM group compared to the HIV/AIDS group, suggesting that patients in the HIV/AIDS+TSM group may develop right heart enlargement. This finding is consistent with evidence that a decrease in IVS and LVPW is associated with right heart enlargement (21). In addition, increased RA and RV sizes may induce right-sided myocardial hypertrophy and heart failure (22). Pulmonary hypertension is a cause of right heart enlargement, but individuals with HIV/AIDS may exhibit RV changes even in the absence of significant pulmonary hypertension (23). Hypoxia is another cause of RV enlargement (24), and since TSM often impairs the respiratory system, RV enlargement in patients with IV/AIDS+TSM may induce hypoxia. The primary role of the RV is to deliver blood into the pulmonary circulation without causing a rise in right atrial pressure and damage to the right ventricle's ejection capability, which can increase RA pressure, leading to an enlargement of the RA (25).

The AECGs of individuals with HIV/AIDS commonly exhibit arrhythmias, prolonged QT intervals, and abnormal ST-T segments, suggesting that HIV can cause electrophysiological abnormalities in cardiomyocytes (4,26). In this study, we also observed notable AECG abnormalities in HIV/AIDS patients. Notably, the HIV/AIDS+TSM group demonstrated a significantly higher prevalence of AECG abnormalities compared to HIV/AIDS patients without TSM co-infection. The HIV/AIDS+TSM group showed profoundly disturbed cardiac electrophysiology, with near-doubling of non-sinus rhythms and ST-T abnormalities, suggesting TSM infection may potentiate HIV-related cardiac electrical instability. The observed electrophysiological abnormalities, emerging prior to detectable cellular damage (27), serve as critical early warning signs of cardiac involvement in HIV/TSM co-infection. These electrophysiological disturbances - particularly the marked ST-T changes and rhythm disturbances - warrant urgent clinical attention given their potential to progress to overt cardiomyopathy if unaddressed. After TSM infection, the host can activate the NLRP3 inflammasome, which may cause damage to cardiomyocytes (28,29). Notably, TSM infection triggers increased IL-1β production through NLRP3 inflammasome activation, a pathway established to cause myocardial ischemia (30,31). This mechanism may underlie the characteristic ST-T abnormalities seen in our AECG findings. Grune et al. (32) reported that cardiac immune cells exert both inflammatory and non-canonical functions, where inflammatory mediators and immune cells can promote arrhythmogenesis through either tissue remodeling or direct cardiomyocyte interactions.

While this study provides the first systematic characterization of cardiovascular abnormalities in HIV/TSM co-infection, several limitations should be acknowledged: our cohort (n=120), though larger than prior TSM cardiovascular studies, may benefit from multicenter validation; the absence of advanced imaging (cardiac MRI, coronary angiography) and extended electrophysiological monitoring (72-h Holter) are diagnostic gaps warranting future investigation, particularly given the technical and clinical constraints of performing invasive angiography in this immunocompromised population; and while we identified characteristic ECG/echocardiographic patterns, the underlying pathophysiology requires elucidation through experimental models. These findings nevertheless establish a foundation for clinical monitoring protocols and motivate mechanistic studies of fungal-mediated cardiovascular pathology.

## Conclusion

Our study revealed that HIV/TSM coinfection produced distinct cardiovascular manifestations characterized by right heart structural changes (including RA/RV enlargement with septal thinning) and pro-arrhythmic electrical abnormalities (particularly ST-T changes and non-sinus rhythms). These findings highlight the need for routine cardiac screening in disseminated talaromycosis, further research into fungal-specific cardiotropic mechanisms, and consideration of echocardiographic monitoring during antifungal therapy to detect and manage these complications.

## Data Availability

The datasets used and analyzed during the current study are available from the corresponding author upon reasonable request.
